# Client-Server Approach for Managing Visual Attention, Integrated in a Cognitive Architecture for a Social Robot

**DOI:** 10.3389/fnbot.2021.630386

**Published:** 2021-09-09

**Authors:** Francisco Martín, Jonatan Ginés, Francisco J. Rodríguez-Lera, Angel M. Guerrero-Higueras, Vicente Matellán Olivera

**Affiliations:** ^1^Intelligent Robotics Lab, Universidad Rey Juan Carlos, Fuenlabrada, Spain; ^2^Grupo de Robótica, Universidad de León, León, Spain

**Keywords:** visual attention, cognitive architectures, social robots, object-based visual attention, robotic cognition, robot vision

## Abstract

This paper proposes a novel system for managing visual attention in social robots. This system is based on a client/server approach that allows integration with a cognitive architecture controlling the robot. The core of this architecture is a distributed knowledge graph, in which the perceptual needs are expressed by the presence of arcs to stimuli that need to be perceived. The attention server sends motion commands to the actuators of the robot, while the attention clients send requests through the common knowledge representation. The common knowledge graph is shared by all levels of the architecture. This system has been implemented on ROS and tested on a social robot to verify the validity of the approach and was used to solve the tests proposed in RoboCup @ Home and SciROc robotic competitions. The tests have been used to quantitatively compare the proposal to traditional visual attention mechanisms.

## 1. Introduction

Mobile social robots incorporate a myriad of sensors and actuators (Kanda and Ishiguro, 2017), for example sonar and LIDAR sensors for obstacle detection, autonomous location and navigation, microphones and speakers for human-robot interaction, and more and more commonly, different types of cameras. Unlike other sensors, the portion of the space that cameras can perceive is limited by their field of vision, which is usually quite narrow compared to the entire space surrounding the robot. Besides, the design of most mobile social robots resembles human morphology. Even those non-humanoid robots place cameras on the robot's head, which is attached to the body by an articulated neck. These actuated cameras overcome the limitations of the narrow field of vision but need to implement an attention management system because it is not possible to simultaneously cover the entire space around the robot. Even if many cameras could be placed in the robot, along with enough computer power to analyze the images, the cognitive system would need to focus on the more relevant elements detected using an attention-managing system. For the sake of this paper, the problem faced is to define where the attention systems have to direct the camera a mobile service robot according to the perceptual needs thereof.

Visual attention systems based on fixed patterns for scanning a scene were the first approaches made, but they have proven to not be very efficient, and more sophisticated approaches, such as the one proposed in this paper, are required (Nguyen et al., 2018). Another aspect to take into account in the evolution of visual attention systems is the integration into complex robotic software architectures which are in charge of selecting the most adequate behavior to fulfill the robot's task. This integration requires the attention system to be modular, parametrizable, and able to share a common way of representing information.

In a previous work, Agüero et al. (2012), a method for managing visual attention, integrated in the cognitive architecture, was initially proposed. In that seminal work, cognitive behaviors indicate their perceptual needs, and the attention system organizes these needs according to their salience. The new approach presented in this paper differs from that work in that the attention system does not arbitrate among behavioral needs, but among elements to be perceived that are indicated by the planning system at the highest level of the cognitive architecture. In order to do so, the system relies on a centralized repository of information that has been implemented as a “knowledge graph.” The robot stores all relevant internal and external knowledge in this repository. The graph contains nodes that represent the elements of the environment, and arcs that indicate the relationships, symbolic or geometric, among them.

The software design of the proposed attention system is modular, allowing specialization in the way different types of stimuli are dealt with. Modularity has been achieved using a client-server systems. This approach is also scalable, meaning that it can be easily expanded with more types of stimuli by adding separate clients. Monolithic approaches to the visual attention systems make them much more difficult to extend.

This implementation has been validated in the RoboCup@Home^1^ and European Robotics League^2^ competitions, which consist of a set of tests which take place in a simulated domestic environment. The performance of each robot is evaluated for tasks focused on assistance or collaboration with humans, which is an excellent way to contrast different research approaches. For all the tasks in these competitions, robots need to visually perceive the scene. Some tasks require the robot to look at a person's face while talking with them or to follow them around a house. For other tasks, the robot must search for objects on a table, or check if it is carrying the correct objects on its tray. All of the task in the competitions require a challenging management of visual attention. In particular, guaranteeing that no interest point remains unattended for a long period of time is one of the most relevant requirements; the time for answering questions about the environment is limited, and the total time for accomplishing the task is also limited. We consider that the maximum time that an interest point remains unattended is the most relevant parameter when comparing different solutions.

In summary, the main contribution made in this paper is the design of the visual attention system. This system is integrated into the cognitive architecture through the knowledge graph, where visual perception requirements are expressed through the creation of arcs between nodes that indicate these requirements.

The remainder of the paper is structured as follows: In section 2 we review the state of the art of visual attention systems, and how different cognitive architectures address their integration. In section 3 we describe the proposed cognitive architecture. Next, we describe the visual attention system. In section 5 real examples of its operation are presented. Finally, in section 6, conclusions are drawn and future work is discussed.

## 2. State of the Art

Visual attention management systems have been a recurrent research topic in mobile robotics. Historically, there have been different approaches to this problem, from basic ones, where segmentation was directly used to focus the attention of the robot, as in Scheier (1997), to ones using methods borrowed from other scientific fields, such as psychology and ethology. For instance, Butko and Movellan (2009) proposed a method of driving a robot that scans scenes based on the model of visual searches in humans. This method predicts scanpaths to maximize the long-term information about the location of the target of interest. In Meger et al. (2008) a combination of a peripheral-foveal vision system, and the attention system that combines bottom-up visual saliency with structure from vision allowed the “Curious George” robot to build a semantic map of the region explored, thereby labeling objects.

The use of visual attention in social robots is widespread. For instance, Kismet (Breazeal and Scassellati, 1999) the famous robotic head which popularized the “affective computing” paradigm, included an attention system capable of directing the robot's eyes toward the areas of interest of an image. These areas of interest, or high salience, were calculated by combining several filters (face detection, color, and movement), which allowed the robot to pay attention to different scene elements. These are the basic questions (Treue, 2003),: *where, what* and *how* such as how to recognize a point of interest, and why it is needed it for scene understanding.

The WABIAN humanoid robot (Hashimoto et al., 2002) was also equipped with an active vision system that directed its gaze toward people, based on images and sound. The work of Wolfe (1994), studying how to determine relevant areas in images, inspired these approaches. This concept of salience is addressed in a multitude of works (Itti et al., 1998; Harel et al., 2006; Hou and Zhang, 2007; Goferman et al., 2012; Grotz et al., 2017), although most focus on which parts of the image are relevant, without spatial information beyond the image. The salience-based approach was previously explored by the authors of this paper, as described in Garcıa et al. (2010), and is still present in the current proposal.

In Bachiller et al. (2008), “Regions of Interest” are used in an image to determine where to direct the camera of a robot. In this case, the robot's active tasks determine the attention zones. Recent works (Stefanov et al., 2019) combine bio-inspired models with Neural Networks to obtain saliency maps, as opposed to spatial areas of the environment. Our approach is not based on the detection of exciting areas in images, but rather areas of space where to direct the robot's camera. We enrich the image-processing with 3-D information. Our attentive system never works on image coordinates but orients itself on the real world. Another relevant difference is that our system is intended to avoid that any interest point identified by the cognitive level could remain unattended for long periods of time.

Integrating the attention system into the cognitive architecture is one of the major problems when using it as a social robot. Some of the systems already mentioned are effective in managing visual attention but are hardly integratable into cognitive architectures.

In Agüero et al. (2012), the authors had proposed a method of visual attention management applied to humanoid robots integrated within the cognitive architecture base on salience. The term salience ceases to be used only for areas of an image and applies to points in space. The salience indicates the need to look at them and increases proportionally to the need to see them. Current behaviors determine this need. In this work, a subsumption architecture (Brooks, 1986), developed for soccer robots (Martin et al., 2010), integrates the attention system. The different execution units that form the behaviors indicate their perceptual needs, and it is the attention system that merges these needs through salience. The current proposal differs from this work in that the attention system does not arbitrate between behavioral needs, but between elements to be perceived by a single behavior.

Cortex is another cognitive architecture, closely related to our own. Its attention system described in Manso et al. (2018) has some similarities to our proposal. The main difference is that Cortex indicates where to find items, instead of determining search points. The system thereby determines which areas of the environment can contain it, thus directing the robot's gaze there. Although similar in many aspects, the system presented in this paper solves the problem of scanning an area and seeing what can be found in it.

iCub robot (Ruesch et al., 2008) applies the concept of EgoSphere, originally by Kawamura et al. (2005). This sphere stores the orientation of the perceived elements to the robot. Saliency and spatial information (angles only) determine the orientation of the robot head. In this case, salience applies to the areas of the self-sphere relevant to the robot. A highly-valued contribution of this approach is sensory fusion. The attention system also adds auditory information to modify the salience of specific areas. Our proposed system is also able to perform advanced spatial reasoning, not limiting the angle of visual stimulation.

Visual attention can be influenced by other sensors. For instance, in Aliasghari et al. (2020), visual attention is used in a social robot to control where the gaze is directed within the context of a group conversation. In order to make this decision, the system uses other stimuli, such as where the people are, where the sound comes from, hand movements, and pointing gestures. It also uses some concepts of proxemic. For instance, it is more natural to look at people who are closer than those further away from the beholder. These stimuli are incorporated into a logical control unit with long-and short-term memory. This control unit decides the neck's movement.

## 3. The Cognitive Architecture for Social Robots

**Figure 1:**
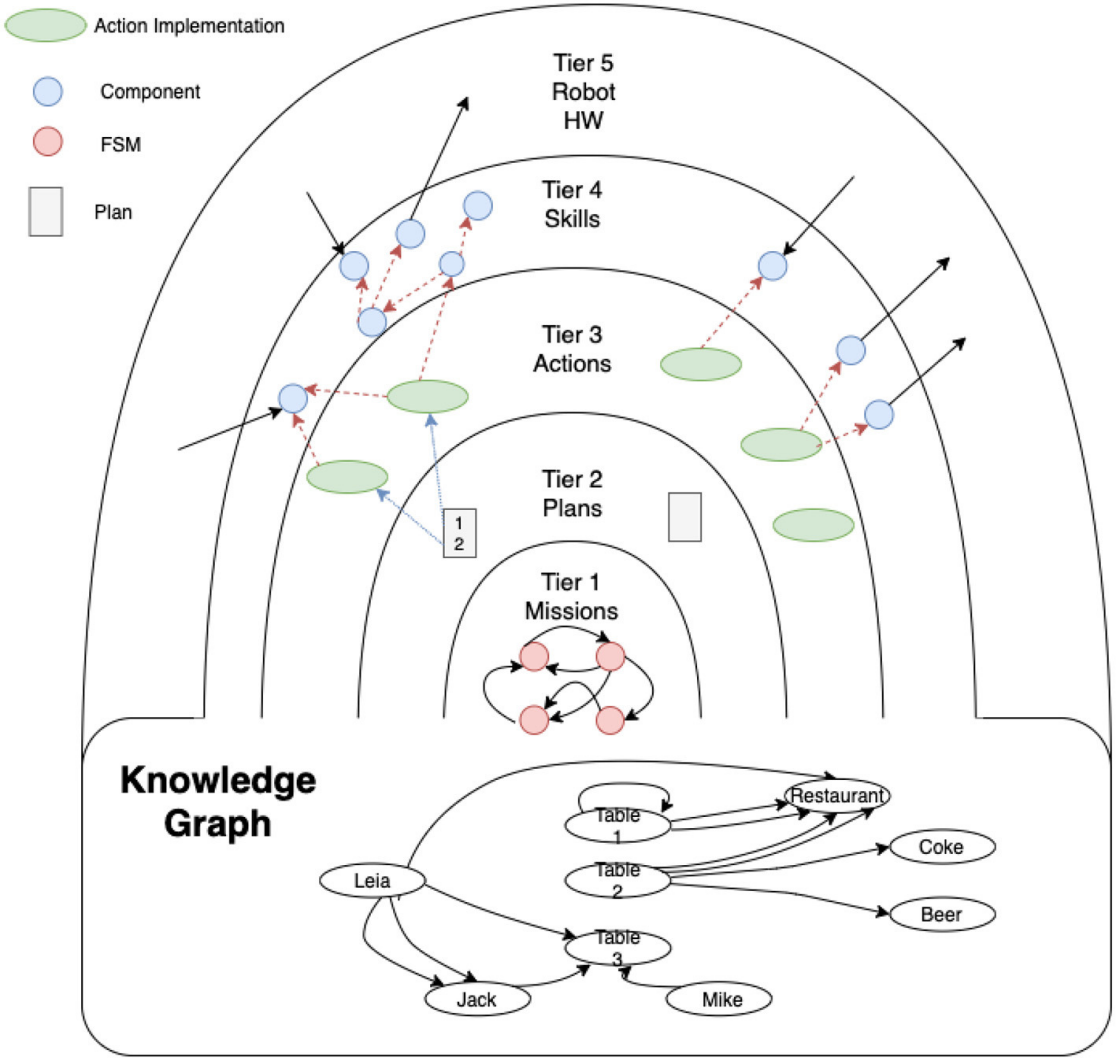
Layered cognitive architecture shown as concentric tiers. The innermost layers (Tier 1) control the mission using a state machine, setting the goals to be achieved by the planning layer (Tier 2). This layer activates the actions that are part of the plan (in Tier 3). Actions can use skills (at Tier 4) to carry out their task. These skills can be perceptual or acting, directly sending information to the actuators, or receiving information from the sensors.

The cognitive architecture, in which the proposed attention system is integrated, is organized in concentric layers, named tiers, as shown in Figure 1. A more detailed description of the architecture can be found in Martin et al. (2020). Describing from the outermost layer to the innermost layer, which can also be considered as a bottom-up description:

- Tier 5 represents the bare metal, the robot hardware and the programming interfaces of the basic controllers of sensors and actuators.
- Tier 4 interacts directly with these controllers to offer a higher level of abstraction in defining the robot's basic *skills*, such as navigating to a place, picking up an object, talking with a person, wandering, detecting objects, etc. The innermost layers use the skills in this layer as primitives.
- Tier 3 is the operational level of the robot. These operations are defined as *actions*. An action uses different skills from tier 4 to accomplish a unit task, e.g., getting the robot to move from one room to another using the skill of navigation. In addition, the robot should take into account whether the door is open, using its perceptual and probably attention skills as well. If the door is closed, the robot will use its manipulation skills to open it and enter the destination room. The actions' implementation defines how the skills are named and which specific parameters (metric destination point, position of the element to manipulate, phrase to speak, etc.) are used. Actions, loops, branches, and sequences can be used to define the control logic for achieving the task.
- Tier 2 is the task manager level where *plans* (ordered executions of actions) are generated. It is based on a symbolic planner which uses PDDL to define what types, symbolic predicates, and actions are used to solve a problem. This knowledge base is accessible by other tiers.
- Tier 1 manages the high-level *mission* of the robot. This level is built using hierarchical state machines which define the different stages of the robot mission at a high level of abstraction. Transitions between states are implemented by consulting predicates in the knowledge base, and the goals to be solved by the planner in Tier 2 are defined in the states.

Tiers 1 and 2 mainly use symbolic information for facing the process of information abstraction, while Tiers 3 and 4 use sub-symbolic information, mainly sensor readings. When a state machine at Tier 1 establishes a goal, the planner at Tier 2 creates a plan using the content of its knowledge base. This plan is built as a sequence of domain actions. The planner delivers the actions at Tier 3 one at a time. Each time an action indicates that it has been successfully completed, the next one is delivered until the plan finishes. If an action ends with an error, it forces a replanning.

As mentioned previously, Tier 3 contains the implementation of the actions defined in the PDDL domain at Tier 2. This level is the bridge between both paradigms. The planner activates actions according to the generated plan. When activated, the planner passes the parameters to the actions (instances of a type). Usually, the action must translate symbols into specific data. For example, a *move* action could receive *kitchen* as a parameter. The action must then obtain the metric coordinate corresponding to the *kitchen* symbol and send it to the navigation module.

In order to manage the information between layers the Knowledge Graph is defined. It stores all the information relevant to the operation of the robot, and is accessible from all the Tiers. This shared representation of data disengages some components from others, especially among different layers. For instance, an action in Tier 3 uses the result of computing a skill in Tier 4 by reading it from the knowledge graph. Tier 1 can also use the symbolic information contained in the graph.

**Figure 2:**
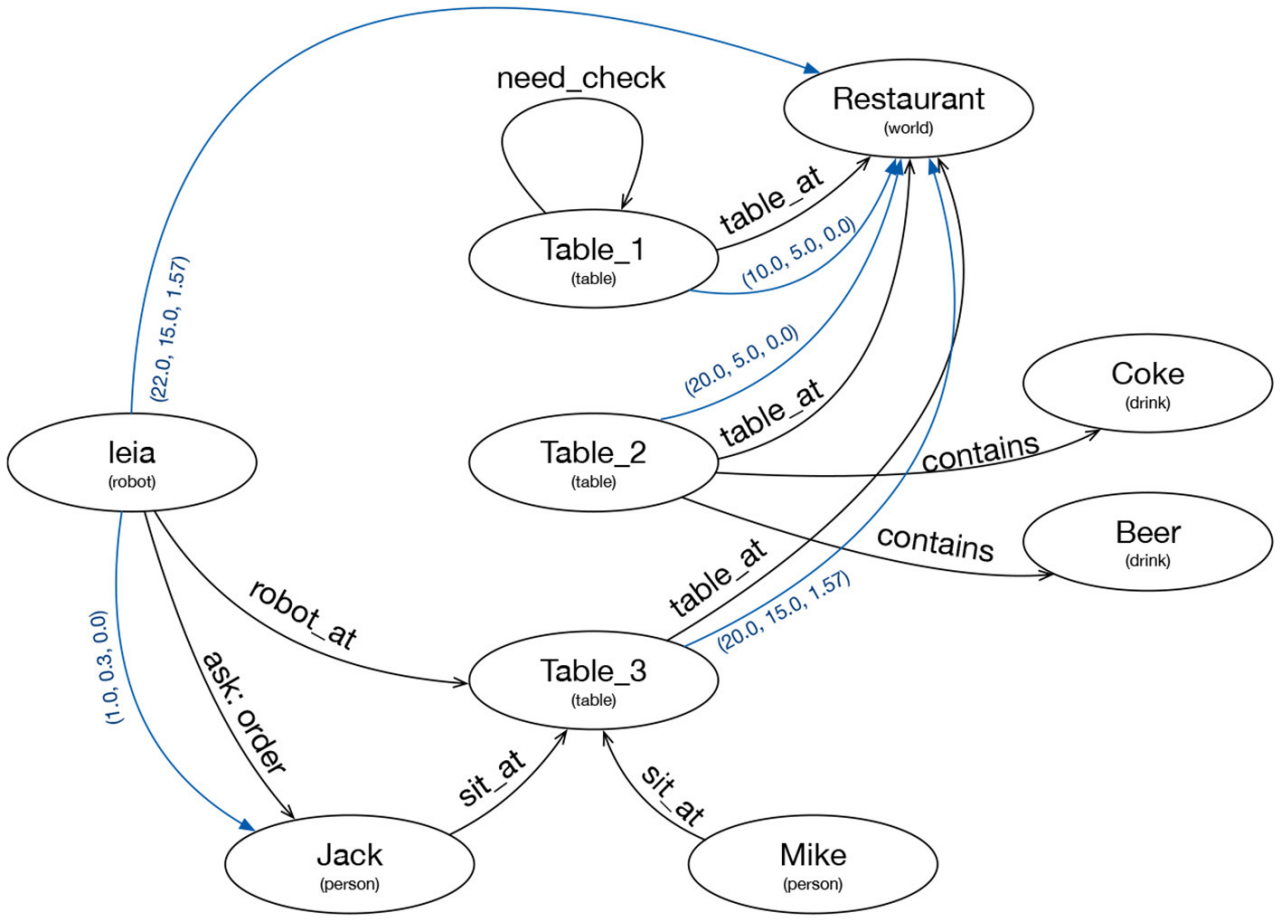
Knowledge Graph representing the internal and external knowledge of the robot. Ellipses represent nodes with an ID and a type. Black lines are text arcs and blue lines are geometric arcs.

The elements of the knowledge graph are nodes and labeled arcs. The nodes represent instances of a specific type. The arches can contain a text, or they can provide a geometric transformation. The visual attention requirements are expressed as arcs of a special type “want_see,” as explained in the next section, where the knowledge graph of Figure 2 is depicted in more detail.

The relationship between the symbolic information at Tier 2 and the global knowledge graph is based on a synchronization process. This process adds nodes to the knowledge graph when the symbolic knowledge base creates instances of a relevant type. It also creates arches when the symbolic knowledge base inserts a relevant predicate. If the predicate has two arguments of related types, the arc connects two nodes with a text corresponding to the predicate. If the predicate has only one argument it is represented as a self arc (*need_check* arc in Figure 2). Updates only go one way, from the symbolic knowledge base to the graph. Updates from the graph to the symbolic knowledge base are not permitted.

ROSPlan (Cashmore et al., 2015) is the planner used in Tier2 and a BICA (Martín et al., 2016) framework was used for the implementation of the actions and skills, as BICA components. A BICA component is an independent process which can declare that it depends on other BICA components. When a BICA component is activated, it automatically activates all its dependencies. When all components which enable a dependency are deactivated, the dependency is deactivated. This mechanism is a simple way to save computation time when the results of certain computations are not being used, and permits different compositions of skills as shown in Figure 2.

## 4. The Attention System

**Figure 3:**
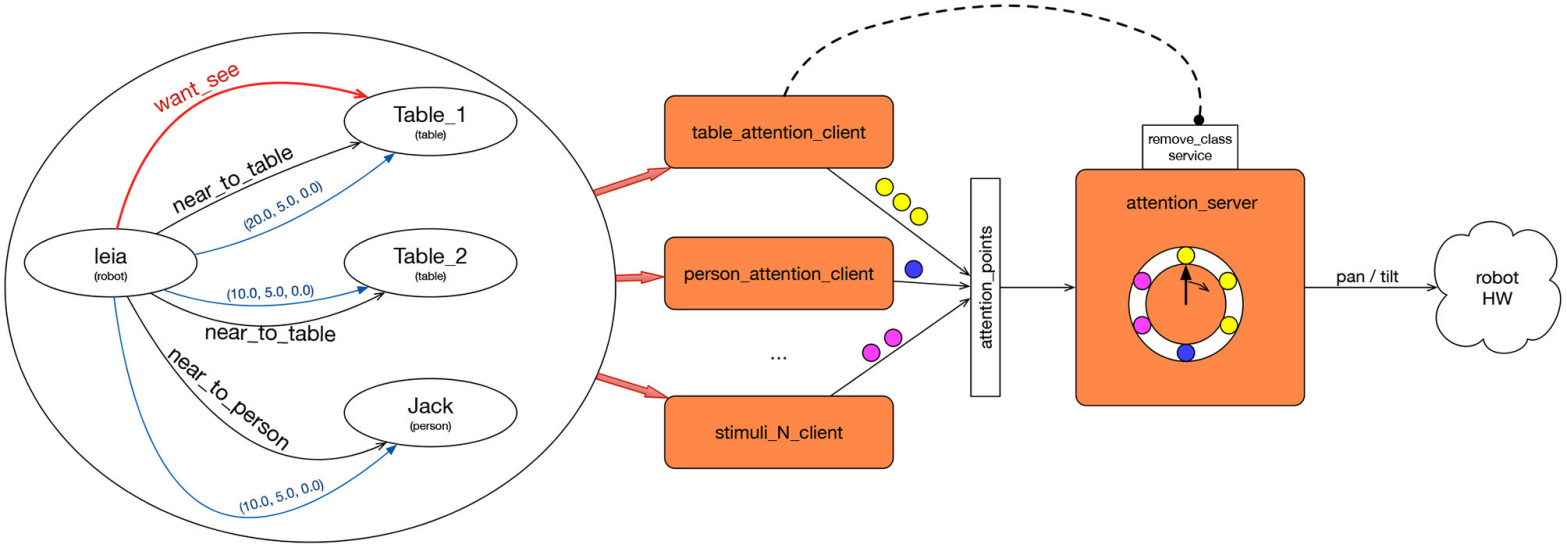
Attention system architecture. The attention clients (orange rectangles in the center) observe the graph **(left)** looking for “want_see” edges. Each client is specialized in one type of goal node for the “want_see” edge. When any of them activates, it sends the attention points to the attention_server **(right)**. The attention_server sends motion commands directly to the neck motors.

The attention system is integrated into the cognitive architecture as a skill in Tier 4. It can be activated by actions in Tier 3 which require attention to different stimuli. Actions set perceptual needs in the knowledge graph by creating wants_see arcs from one robot node to another, as shown by the red arrow in the left part of Figure 3. Other skills in Tier 4 can also add arches requesting attention, as well as in the innermost tiers if it is considered necessary.

The attention mechanism is built as a client-server system, as shown by the orange boxes in Figure 3. The three clients in the figure send *attention points* to the attention server. The *attention server* sends motion commands to the robot's actuators in order to direct the camera to a position in the space. Its main task is to select the next pose to look at among all the requests received and the length of time to maintain that position in the robot's field of view. The attention clients make requests to the server by sending attention points.

Each attention point sent to the server is labeled with the stimulus type and the id to perceive. A client can communicate to the server that it no longer requires attending to a specific type and/or instance. The server then removes these points from its list.

There are as many clients as types of stimulus to deal with. If the robot wants to perceive a table, it can mean that it is interested in either scanning the entire surface, or in determining the existence of the table itself. Different clients should be built for each one. For example, if interested in the objects on the table, the attention points will cover the table's surface, apart from assuming that the table is a static element of the environment. If the robot wants to perceive a small object, there will be a single attention point in the center of the object, if already detected. If the object has not been detected yet, the points will be placed where it is more likely that the object could be. Each type of stimulus requires a custom client specialized in perceiving it adequately. For example, while perceiving a person, it can be enough to look at his face, but these are dynamic points.

Attention clients scrutinize the Knowledge graph in case their participation is required, that is, they look for the existence of wants_see arcs from one node to an element of the type that this client manages. If so, it generates a set of attention points with geometric information that indicates where to direct the robot's camera to perceive the stimulus.

In the case of Figure 3, there are nodes of types *robot, table*, and *person*. There are also geometric and symbolic arches. Several processes, named [stimulus]_attention_client one for each type of node which the robot can pay attention to, are shown in the center. Each one is aware of the changes in the graph. In this case, table_attention_client should be active because there is an arc from a node of type robot to a node of type table. When active, table_attention_client sends a set of attention_points in the frame of Table_1 to check.

Furthermore, the attention_server receives the attention points of all the clients and iterates among each one of them. The attention_server maintains a list with all the attention points received. For each point, it transforms it into a coordinate related to the axes of the robot, and generates a pan and a tilt that it sends to the motors of the robot's neck, visiting each point for a few seconds.

The attention module is not responsible for image detection, only for looking there. If an action requires detecting objects on a table, this action must activate both the attention and the module that perceives the objects in the image. When an object is detected in the image, this is written down in the graph, thereby allowing eliminating the corresponding attention arcs from the action.

Attention clients send the points of attention of each one of the elements to attend to (small circles of Figure 3) to the server. Messages sent to the server contain the class and identifier of the element. In addition, they contain a vector of 3D points. For each point, we specify the reference axis (frame_id) of its coordinates.

The attention server receives the set of new points, NP, sent by the clients. The server stores the points received in a list. Each point, *p*, on the list P that the server stores contains the following fields:

- point_id: The attention server must be able to attend to requests to eliminate points of attention, either by specifying an entire class or just one instance of a class. This field contains an identifier class.instance_id.n, where *n* is the i-th point of attention of one of the elements. In this way, it is easy to determine the points that belong to each class and instance.
- point: The point coordinates, stamped with its frame_id and time.
- tilt and pan: The axes of reference of the points can move with regard to the robot. That means that points could be coordinates of a global map, and the robot could be moving, or points could be coordinates of the robot arm, and the robot could be moving its arm.
- epoch. Attention cannot be paid to one point again until the rest of the points have been attended to. Epoch represents the current iteration of the attention system. Attentions server does not attend to a point if there is another point with a lower epoch value. Each point attended increases its epoch by one.

The attention server calculates the *pan* and *tilt* values to send to the robot's neck actuators, calculated from P. *pan*_*t*−1_ is the current pan value, and *pan*_*t*_ is the new pan value to send to the actuators.

This algorithm is summarized in Algorithm 1 and is based on these three rules:

1. The robot cannot handle an attention point again until after handling the other attention points.
2. The next attention point is the point that implies the least head movement.
3. If the next attention point is already in the fovea, it is considered handled.

**Algorithm 1:** Attention Server algorithm.

1: **while** *robot*_*operation* **do**
2: **for all** $p_{i}\in \mathrm{NP}$ **do**
3: $p_{i}^{epoch}=p_{j}^{epoch}$, where $p_{j}=last\left(P\right)$
4: $P\leftarrow p_{i}$
5: **end for**
6: **for all** $p_{i}\in P$ **do**
7: $p_{aux1}=RT^{4\times 4}\left(p_{i}^{frame}\to pan\_frame\right)*p_{i}$
8: $p_{aux2}=RT^{4\times 4}\left(p_{i}^{frame}\to tilt\_frame\right)*p_{i}$
9: $p_{i}^{pan}=\arctan\left(p_{aux1}^{y},p_{aux1}^{x}\right)$)
10: $p_{i}^{tilt}=\arctan\left(p_{aux2}^{z},p_{aux2}^{x}\right)$)
11: **end for**
12: sort(P)
13: **repeat**
14: p=first(P)
15: *p*^*epoch*^ = *p*^*epoch*+1^
16: **until** is_in_fovea(*p*)
17: pan_t = *p*^*pan*^
18: tilt_t = *p*^*tilt*^
19: send_command_to_neck(*pan*_*t*_, *tilt*_*t*_)
20: *t*_*flight*_ = flight_time(*pan*_*t*−1_, *pan*_*t*_, *tilt*_*t*−1_, *tilt*_*t*_)
21: *t*_*in*_*point*_ = 1.0 s
22: wait(*t*_*flight*_ + *t*_*in*_*point*_)
23: **end while**

In more detail, the algorithm works as follows:

- Lines 2-5 show how the server incorporates the new points NP received from clients to the list P of attention points.
- Lines 6-11 recalculate the pan and tilt of each point before sorting. Many points could be defined in frames that have changed with regard to the robot's neck. If we define points in map frame, their new pan/tilt values depend on the robot displacement and the localization.
- Line 12 sorts P using the operator “<” defined as:
  $$p_{i}<p_{j}=\{\begin{array}{l} \text{if }p_{i}^{epoch}<p_{j}^{epoch},\\ \text{or}\\ \text{if }p_{i}^{epoch}=p_{j}^{epoch}\text{and }j(p_{i})<j(p_{j}) \end{array}$$
  where,
  $$\begin{array}{l} j\left(p\right)=|p^{pan}-pan_{t-1}|+|p^{tilt}-tilt_{t-1}| \end{array}$$
  From now on, the most appropriate points to pay attention to are at the top of the list.
- Lines 13–16 select the point *p* to attend on the 13–16. Starting at the beginning of the list, we take the first one that is not in the fovea.
- *p* point contains the new pan and tilt values. After sending them to the actuators, the waiting time before sending other values to the actuators must be calculated. This waiting time depends on two time periods. The first one is the duration of positioning in the new pan/tilt values (line 20). The second one is the time in which the robot maintains attention to a point. It is convenient for the robot to stop at a point for a short moment. The image could be moving or degenerating its processing. We consider a second to be an appropriate value.

Our attention system's design allows us to efficiently attend to visual stimuli (objects, people, areas, etc.) since it personalizes the attention for each stimulus type. The system is also scalable: a new kind of stimulus to attend to requires only creating an attention client that defines the points of attention in the stimulus's reference axis. In the next section we will show the experiments carried out to validate our proposal, a simple way to save computation time when the results of certain computations are not being used and which allows different compositions of skills as shown in Figure 2.

## 5. Experimental Validation

This section describes the experiments carried out with a real robot to evaluate the validity of our proposal. The main feature of an attention system is attending to the relevant areas of robot operation. In order to determine what the advantages of the proposed system are, two other classical approaches have also been implemented:

1. Slow scan across the environment around the robot. This mechanism would be activated whenever the robot wants to perceive something, moving the robot's neck in a fixed pattern.
2. Selection of the point of attention, *p*_*i*_, from the list P using round-robin without ordering the points to optimize the movement of the robot's neck.

The metrics used to compare this proposal vs. these other two approaches are:

- The percentage of time that the robot is attending relevant areas.
- The time to cover all relevant areas.
- The amount of energy used to cover all relevant areas.

The correctness of the detections has not been included as criterion for the experimentation because it depends on other modules in charge of perception. Neither has a quantitative analysis of the integration of the attention system in the cognitive architecture been included because this analysis can only be done qualitatively. In our system was validated by integrating it into the software of the SciRoc Robotics competition.

**Figure 4:**
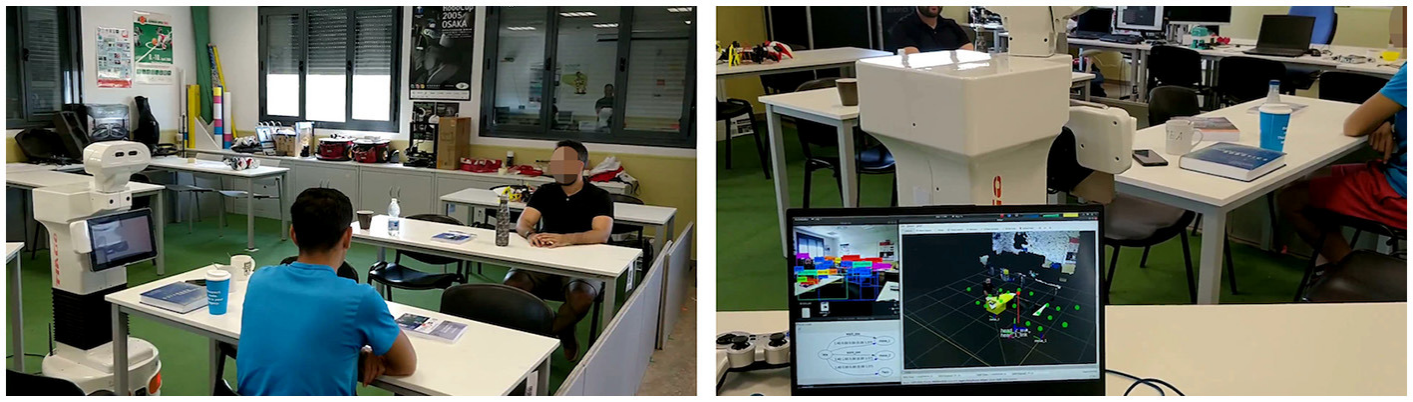
Left image: Experimental setup. Right image: Laptop screen showing the objects being perceived (left part of the screen and their spatial location).

The SciRoc competition environment Figure 4 was reproduced for the evaluation. It simulates a restaurant in which the robot should check how many persons are sitting at the tables and which objects are on the tables. The robot is in front of one of the tables (mesa_1), and to its left there is another table (mesa_2). There are several objects to be detected on the tables, and two people sitting, one at each table. The setup of this experiment can be seen in this video ^3^.

**Figure 5:**
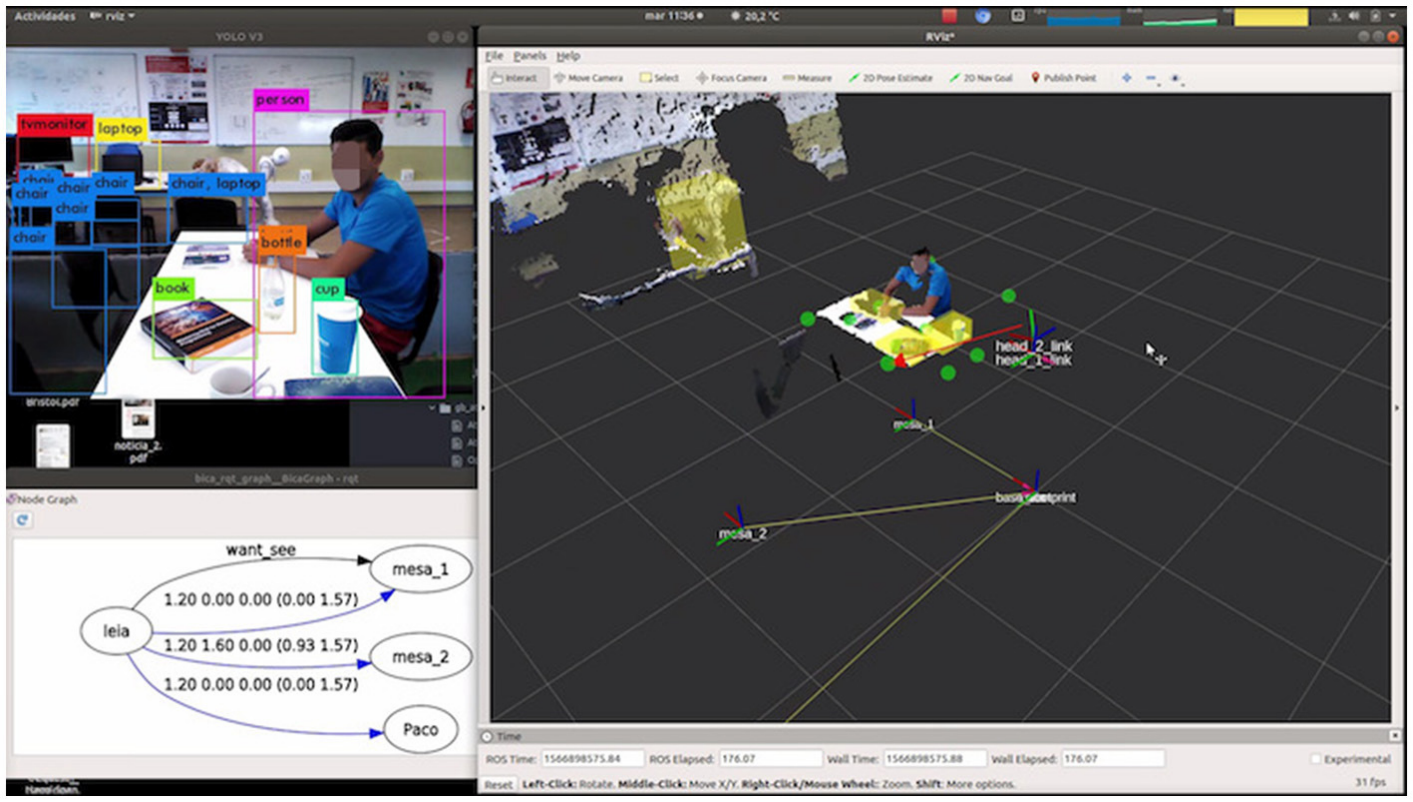
Visual debugging of detections (left upper image), knowledge graph (left bottom figure), attention points (green circles), and attending point (red arrow) in right part of the figure.

The robot knows *a priori* its relative position to the table. This information is introduced in the knowledge graph (Figure 5). A table_attention_client was implemented which establishes 10 points of attention per table: 6 on the surface and 4 in positions where there could be people. Attention points can be seen in Figure 5 depicted as green circles.

A skill that adds “want_see” arcs in the graph from the robot to the tables was specifically designed for this setup. Every 30 s, the “want_see” arc is added to or deleted from table_2 (*mesa_2* in the figure). When both arches are active, there are two points of attention on each table. Initially, we activate attention for two tables, so there are 20 attention points. At time 30, we remove an arc, so the robot is attending to only one table, and there are 10 points' the cycle loop restarts at time 60.

The attention mechanisms were compared with two alternatives previously mentioned:

- *Round Robin*: the robot evaluates attention points in the order in which they are stored on the server, which can be expressed as a new operator <′ defined as:
  $$\begin{array}{l} p_{i}{<}^{\prime }p_{j}\text{ if }p_{i}^{epoch}<p_{j}^{epoch} \end{array}$$
- *Scan*: The robot continuously moves its neck to cover the robot's environment. This approach was optimized so that it only scans the areas where there are points of attention. Before scanning, it calculates the range of pan/tilt angles based on the current points of attention.

**Figure 6:**
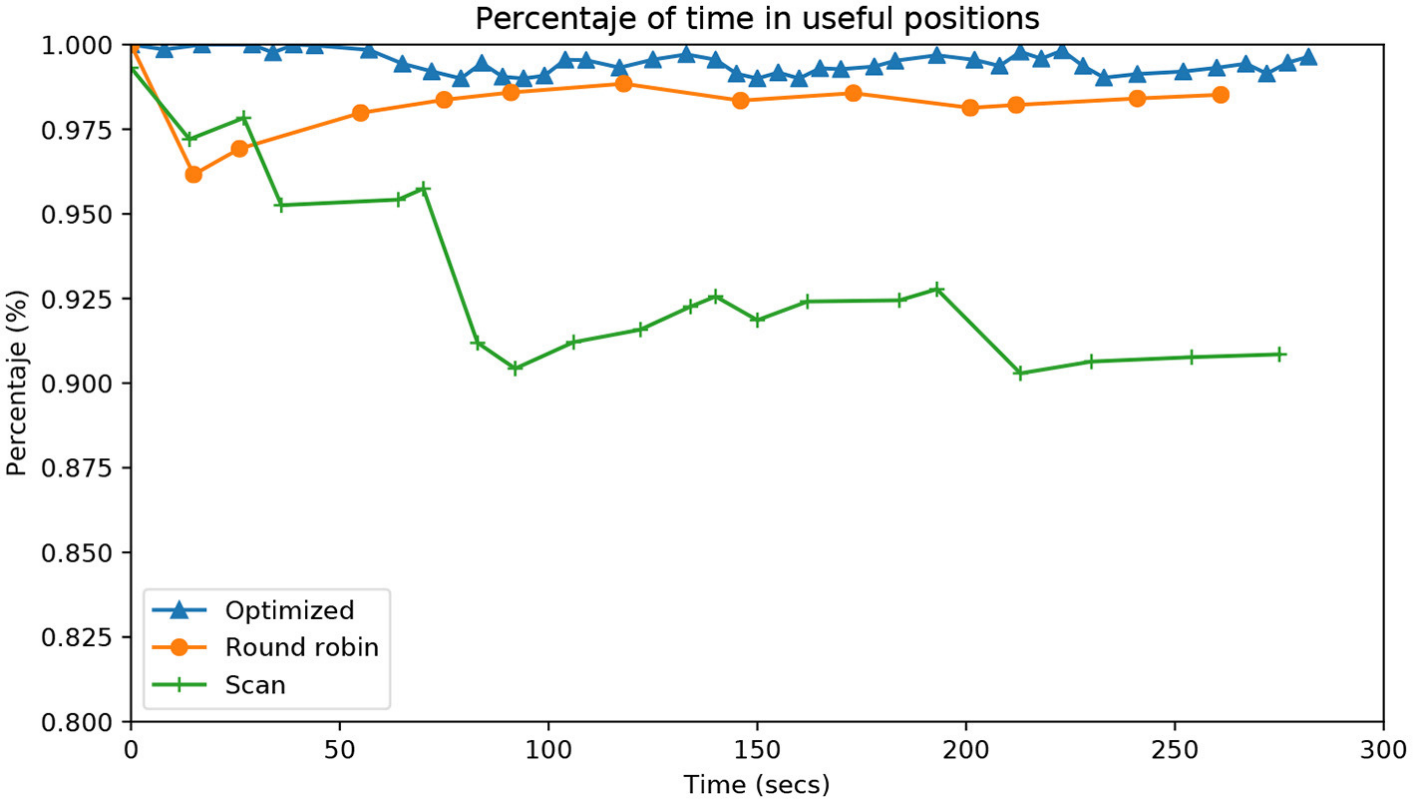
Evolution during the entire time of the experiment (*X*-axis) as a percentage of time (*Y*-axis) that there is any attention point in the fovea (central part of the image). Each line represents a different algorithm, comparing our contribution (Optimized) with respect to the two usual algorithms of attention (Simple scanning and Round robin).

Figure 6 shows the accumulated percentage of time that the robot has any attention point in the fovea. The fovea is the central area of the image, half the size of the total image. The marks on the lines of each approach indicate when each epoch is completed. As expected, the Scan approach is significantly worse than the others.

**Figure 7:**
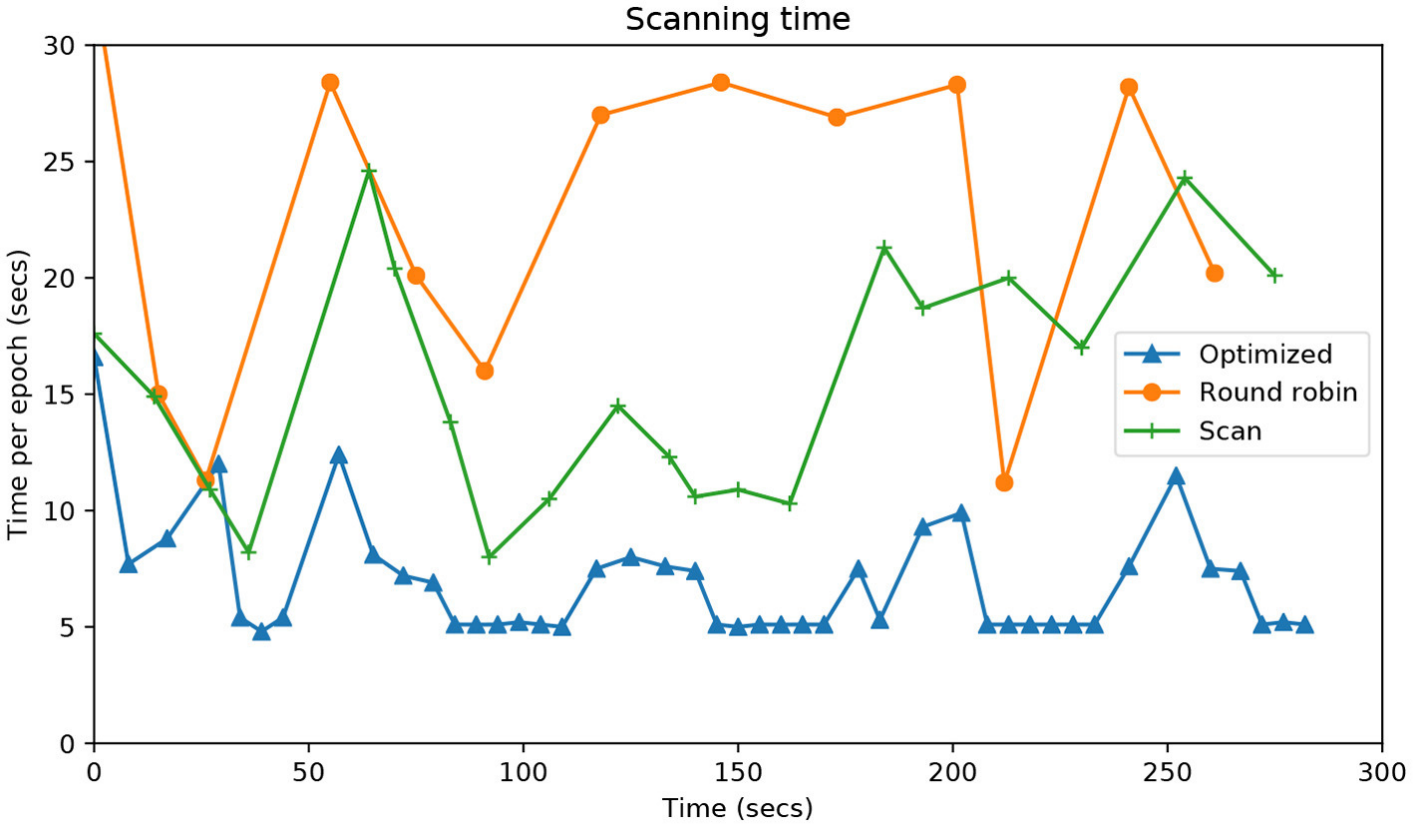
Evolution during the entire time of the experiment (*X*- axis) vs. the time in seconds (*Y*-axis) that it takes each algorithm to visit all the attention points.

Figure 7 shows the time it takes for each approach to complete an epoch, that is, to visit each of the points of attention. The system proposed shows times of around 5 s when there is only one table active. In the case of two tables, the time only exceeds 15 s once. The Round Robin method takes more than double the time in virtually all epochs. In any case, these results are much better than those of the Scan method.

**Figure 8:**
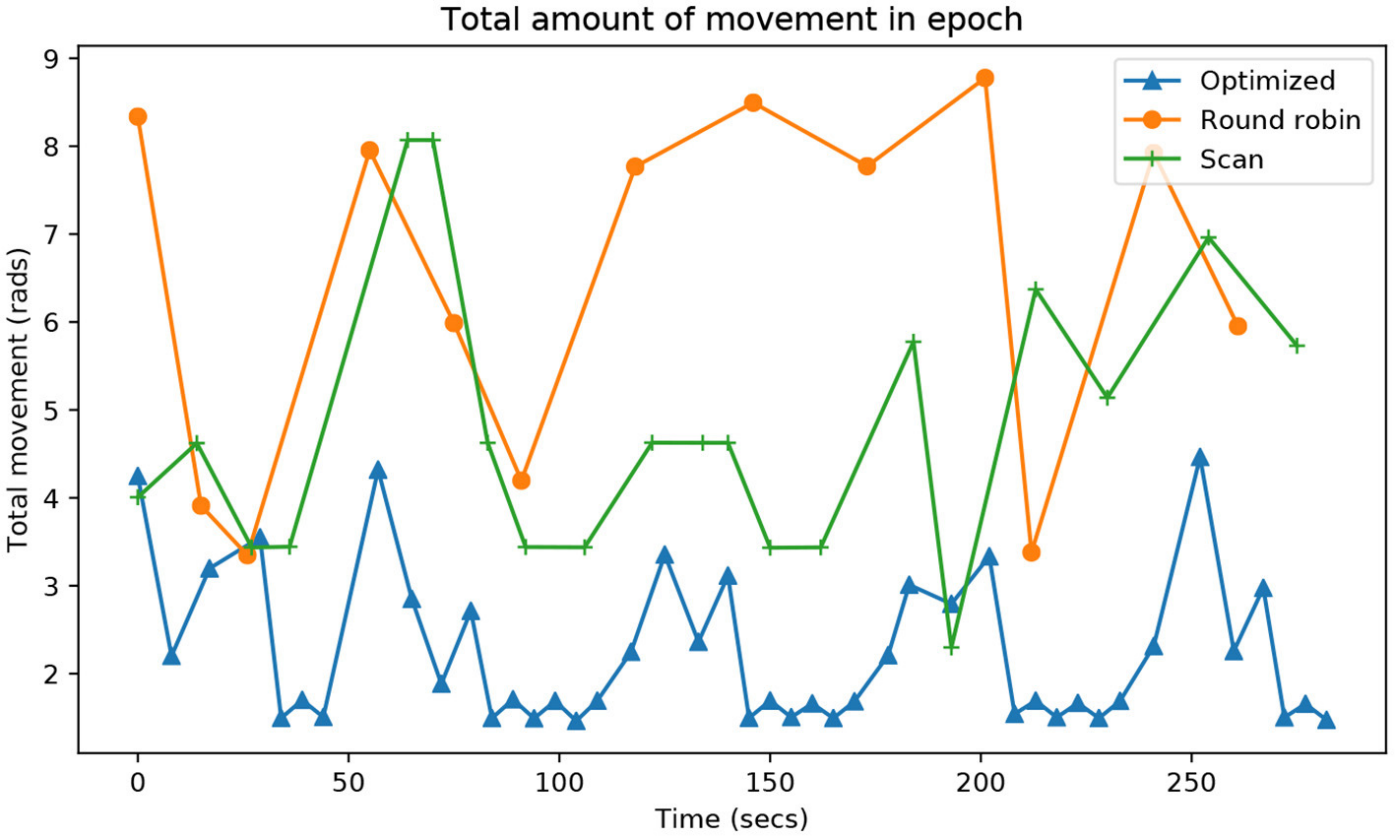
Evolution during the entire time of the experiment (*X*-axis) of the accumulated neck rotation, in radians (*Y*-axis), that it takes each algorithm to visit all the attention points.

The last indicator is the energy required in each epoch. As it is difficult to obtain energy measurements, the difference between the current angle and the commanded angle was measured. The smaller the displacement of the head, the lower the energy required to visit a point of attention. Figure 8 shows that the proposed system is also, by far, the one that preserves the most energy to complete each epoch.

**Figure 9:**
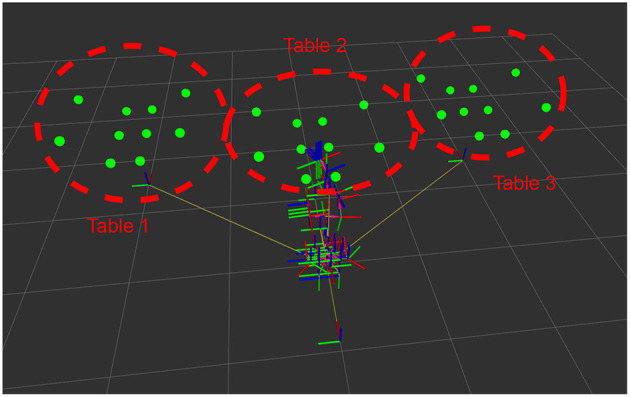
Attention points (green circles) corresponding to the setup for the second experiment, with the position of the robot (denoted by the axes of its actuators). In this case, the attention points were located on three tables.

We carried out a second experiment to measure the time it takes for each algorithm to return to an attention point. Figure 9 shows the distribution of the points of attentions (green dots) and the coordinates transformation from the three tables to the robot using the ROS tf visualization tool. The robot is in front of three tables, each one with the same attention points. As in the previous experiment, we consider that the robot deals with a point of attention when it is in the fovea. An algorithm is considered best if it does not allow points to be unattended for a long time.

We have carried out multiple runs of the three algorithms attending one, two and three tables. In each case, we measured the time that a point of interest is unattended. Each trial lasts 2 min, and the results of the experiment are shown in Table 1.

**Table 1:** Results of the second experiment, showing the time (in seconds) to deal with an attention point, and the number of points attended.

	**1 Table**			**2 Tables**			**3 Tables**		
	**Opt**	**RR**	**Scan**	**Opt**	**RR**	**Scan**	**Opt**	**RR**	**Scan**
Mean	6.38 s	5.95 s	9.44 s	9.11 s	6.70 s	12.01 s	9.42 s	7.69 s	14.50 s
Stdev	4.40 s	6.90 s	7.76 s	5.71 s	6.84 s	0.83 s	5.47 s	7.69 s	13.20 s
Median	3.99 s	3.09 s	6.29 s	7.94 s	4.29 s	6.19 s	8.99 s	4.69 s	6.20 s
**Max**	**18.89 s**	28.49 s	31.09 s	**28,39 s**	37.39 s	37.89 s	**25.69 s**	50.09 s	41.39 s
Points	131	134	90	192	265	135	285	345	191

The table's analysis reveals that the Round Robin algorithm yields the best times in the mean and the median. Also, more points are served in the 2 min that each trial lasts. The numbers are very similar to the algorithm that optimizes attention, although it deals with fewer points during these 2 min. Still, the maximum time a point has waited to be observed is much longer with the Round Robin algorithm, which is the critical factor that we tried to minimize with our proposal. The scan algorithm, which is the baseline in this work, has the worst statistics, showing that our proposal significantly improves a robot's attention.

In order to illustrate the criticality of the maximum time parameter, it has to be noted that in the competitions, the time that the robot is inactive is very limited. For instance, the rulebook^4^ of the RoboCup competition states that 30 s of inactivity disqualifies a robot. In the same way, the maximum time for each trial is also limited.

## 6. Conclusions

This paper has presented a visual attention system integrated into a cognitive architecture. This attention system calculates the head movements necessary to perceive the elements of the robot's environment. The cognitive architecture integrates the attention system as a robot skill. Perceptual needs are expressed in a degree of knowledge, adding arcs that indicate this. The attention system is aware of these attention arcs. The way of attending to an element of the environment depends on the objective of this arc.

The experiments carried out show that the system proposed improves conventional systems based on scanning the environment. The robot's gaze always goes to relevant areas without wasting time in areas where the searched items cannot be found. Attention to these areas is always given using the lowest possible energy and stabilizing the image in a position to perform the detection with sharp images.

We have tested this approach on a real Tiago robot. We have proven its validity in one of the tests of the SciRoc competition. To validate our approach, we have implemented two representative attention methods. In this experimentation, we have shown that our approach improves the other methods according to the maximum time, which is the main factor in this problem and has been highlighted in Table 1.

Finally, as a further work, we think that the energy consumed by each method should be analyzed, as well as the relevance of the order of the points in the RR method, fixed by the designer in this method.

## Data Availability Statement

The original contributions presented in the study are included in the article/supplementary material, further inquiries can be directed to the corresponding author/s.

## Author Contributions

FM and FR-L were the main designers of the software architecture. JG and AG-H were responsible for the experimentation, and VM was the coordinator of the team and contributed in the design of the cognitive architecture. All authors contributed to the article and approved the submitted version.

## Funding

This work has been partially funded by Ministerio de Ciencia, Innovación y Universidades through grant RTI2018-100683-B-I00.

## Conflict of Interest

The authors declare that the research was conducted in the absence of any commercial or financial relationships that could be construed as a potential conflict of interest.

## Publisher's Note

All claims expressed in this article are solely those of the authors and do not necessarily represent those of their affiliated organizations, or those of the publisher, the editors and the reviewers. Any product that may be evaluated in this article, or claim that may be made by its manufacturer, is not guaranteed or endorsed by the publisher.

## Footnotes

^1^ http://www.robocupathome.org/

^2^ https://www.eu-robotics.net/robotics_league/

^3^ https://youtu.be/IyCyx_HfdrE

^4^ http://www.robocupathome.org/rules
